# Mitochondrion-Localized SND1 Promotes Mitophagy and Liver Cancer Progression Through PGAM5

**DOI:** 10.3389/fonc.2022.857968

**Published:** 2022-03-31

**Authors:** Shiwei Liang, Chuxu Zhu, Caixia Suo, Haoran Wei, Yingxuan Yu, Xuemei Gu, Liang Chen, Mengqiu Yuan, Shengqi Shen, Shiting Li, Linchong Sun, Ping Gao

**Affiliations:** ^1^ School of Medicine, South China University of Technology, Guangzhou, China; ^2^ Guangzhou First People’s Hospital, School of Medicine, South China University of Technology, Guangzhou, China; ^3^ Guangdong Cardiovascular Institute, Guangdong Provincial People’s Hospital, Guangdong Academy of Medical Sciences, Guangzhou, China; ^4^ Chinese Academy of Sciences (CAS) Key Laboratory of Innate Immunity and Chronic Disease, Hefei National Laboratory for Physical Sciences at Microscale, Innovation Center for Cell Signaling Network, School of Life Science, University of Science and Technology of China, Hefei, China

**Keywords:** SND1, PGAM5, mitophagy, tumor growth, hepatocellular carcinoma

## Abstract

Staphylococcal nuclease domain-containing protein 1 (SND1) is an evolutionarily conserved multifunctional protein that functions mainly in the nucleus and cytoplasm. However, whether SND1 regulates cellular activity through mitochondrial-related functions remains unclear. Herein, we demonstrate that SND1 is localized to mitochondria to promote phosphoglycerate mutase 5 (PGAM5)-mediated mitophagy. We find that SND1 is present in mitochondria based on mass spectrometry data and verified this phenomenon in different liver cancer cell types by performing organelle subcellular isolation. Specifically, The N-terminal amino acids 1-63 of SND1 serve as a mitochondrial targeting sequence (MTS), and the translocase of outer membrane 70 (TOM 70) promotes the import of SND1 into mitochondria. By immunoprecipitation-mass spectrometry (IP-MS), we find that SND1 interacts with PGAM5 in mitochondria and is crucial for the binding of PGAM5 to dynamin-related protein 1 (DRP1). Importantly, we demonstrate that PGAM5 and SND1-MTS are required for SND1-mediated mitophagy under FCCP and glucose deprivation treatment as well as for SND1-mediated cell proliferation and tumor growth both *in vitro* and *in vivo*. Aberrant expression of SND1 and PGAM5 predicts poor outcomes in hepatocellular carcinoma (HCC) patients. Taken together, these findings establish a previously unappreciated role of SND1 and the association of mitochondrion-localized SND1 with PGAM5 in mitophagy and tumor progression.

## Introduction

Mitochondria are highly dynamic, double-membrane subcellular organelles that perform important functions in the homeostasis of health and diseases. Under conditions involving DNA damage or hypoxic treatment, the p53 protein localizes to mitochondria and forms a specific inhibitory complex with the protective antiapoptotic members Bcl-xL and Bcl-2 to promote the p53-dependent apoptosis (1–3). PTEN was found to sustain ROS production and mitochondria-dependent apoptosis through mitochondrial localization (4, 5). Following phosphatidylinositol 3-kinase activation, AKT is transported to mitochondria (6), and then modulates the reprogramming of somatic cells and mitochondrial apoptosis (7, 8). Staphylococcal nuclease domain-containing protein 1 (SND1), also known as p100 or TSN, is an evolutionarily conserved protein that plays multifaceted roles by acting as a transcriptional coactivator (9) or is involved in the processing of precursor messenger RNA (10), miRNA decay (11), and ubiquitination and degradation (12) through localization to the cytoplasm and nucleus during tumorigenesis. Recently, SND1 was reported to play essential roles in breast cancer metastasis (13, 14); however, whether SND1 localizes to mitochondria and regulates cell proliferation and tumor progression through mitochondrial-related functions is largely unknown.

Mitochondria perform important functions in energy metabolism, mitochondrial dynamics, apoptosis, and mitophagy. SND1 is a substrate for caspase-3 during apoptosis and is a conserved component of the programmed cell death pathway (15). Wan et al. found that disruption of the metadherin (MTDH)-SND1 complex sensitizes breast cancer cells to replication stress-induced apoptosis (16, 17), and Shen et al. demonstrated that disruption of the MTDH-SND1 complex by small-molecule inhibitors or compounds enhances the sensitivity of cancer cells to anti-programmed cell death protein 1 therapy, thus suppressing breast cancer progression and metastasis (13, 14), but it remains unclear whether SND1 regulates mitophagy. Mitophagy, the selective degradation of dysfunctional or damaged mitochondria under stresses such as nutrient limitation, hypoxia, and chemical inducers, is a sophisticated system conserved from yeast to human that regulates mitochondrial quality and participates in quantity control to maintain mitochondrial fitness. Receptor (BNIP3, FUNDC1, PGAM5)-mediated mitophagy and ubiquitin (PINK1, Parkin)-mediated mitophagy are the two major forms of mitophagy (18). It should be noted that, although mitophagy is dysregulated in cancer patients, its roles in tumorigenesis largely depend on the cellular context. For instance, ROS-driven mitophagy differs in different stages of tumor development, mitophagy inhibits early tumorigenesis by maintaining normal cellular metabolism, but promotes tumor development by improving cell tolerance for better adaption to the tumor microenvironment at the late stage (19). Thus, further elucidation of the relationship between SND1 and mitophagy will expand our understanding of the noncanonical function of SND1 and its roles in tumorigenesis.

Here, we found that SND1 localizes to the mitochondria with the assistance of its N-terminal amino acids 1-63 and TOM 70. Additionally, increased mitochondrial SND1 is observed under stress conditions. Mitochondrion-localized SND1 promotes mitophagy by directly binding to PGAM5. PGAM5 and the MTS of SND1 are required for SND1-promoted mitophagy, cell proliferation, and tumor growth. Finally, our results indicate that SND1 and PGAM5 are two potential prognostic markers of HCC patients.

## Materials and Methods

### Cell Lines and Cell Culture

Human liver cancer cell lines (Hep3B, PLC and HepG2) and HEK293T were cultured in Dulbecco’s modified Eagle’s medium (DMEM) supplemented with 10% fetal bovine serum (FBS) and 1% penicillin/streptomycin. All cells were kept in a humidified incubator at 37°C and 5% CO_2_. FCCP (T6834) was purchased from Topscience Biotech Co., Ltd.

### Plasmids and Stable Cell Line Construction

Short hairpin RNA (shRNAs) targeting SND1 were commercially purchased (Sigma-Aldrich). shRNAs targeting PGAM5 and the 3′-UTR of SND1 were constructed in the PLKO vector. SND1, PGAM5, TOM20, TOM70, MTS-GFP, and DRP1 were subcloned into the pSin-3×Flag vector or pSin-HA vector (Addgene). Each lentiviral plasmid was co-transfected with plasmids encoding Δ8.9 and VSVG into HEK293T packaging cells using PEI (polysciences). Viral supernatant was collected 48 h posttransfection and filtered. The transduced cells were infected with the produced lentivirus in the presence of polybrene and selected with puromycin to establish stable cells. The primer sequences used in these experiments are listed in online **Supplementary Tables 1** and **2**.

### Immunoblotting

For immunoblotting, proteins were extracted from cells/tumor samples by using RIPA buffer (50 mM Tris-HCl, pH 8.0, 150 mM NaCl, 5 mM EDTA, 1% NP-40 and 0.1% SDS) supplemented with protease cocktails and 1 mM phenylmethylsulfonyl fluoride (PMSF). The supernatant was collected after centrifugation at 13,000 rpm for 10 min at 4°C. Protein concentration was measured using the Bradford assay kit. Equal amounts of proteins were fractionated by SDS-PAGE. Signals were detected using Western ECL Substrate (Tanon). Primary antibodies against the following proteins were used: SND1 (1:2,000, ab65078, Abcam), PGAM5 (1:1,000, sc-515880, Santa Cruz Biotechnology), TOM20 (1:1,000, 11802-1-AP, Proteintech), TOM70 (1:1,000, 14528-1-AP, Proteintech), COX4 (1:1,000, 11242-1-AP, Proteintech), TIM23 (1:1,000, sc-514463, Santa Cruz Biotechnology), MFN1(1:1,000, 13798-1-AP, Proteintech), LC3 (1:1,000, NB100-2220, Novus Biologicals), Phospho-DRP1 (Ser637) (1:1,000, 4867S, CST), DRP1 (1:1,000, 12957-1-AP, Proteintech), Lamin B1 (1:2,000, 12987-1-AP, Proteintech), Tubulin (1:5,000, 66031-1-Ig, Proteintech), β-Actin (1:2,000, 66009-1-Ig, Proteintech), GAPDH (1:5,000, 60004-1-Ig, Proteintech), TFAM (1:2,000, 22586-1-AP, Proteintech), Flag-M2 (1:2,000, F1804, Sigma-Aldrich), HA (1:1,000, 51064-2-AP, Proteintech), HA-HRP (1:1,000, 2999S, CST), GST (1:5,000, 10000-0-AP, Proteintech), GFP (1:4,000, 50430-2-AP, Proteintech), Calnexin (1:5,000, 10427-2-AP, Proteintech), HRP-conjugated anti-rabbit and anti-mouse secondary antibodies (Bio-Rad) were used.

### Immunoprecipitation

For immunoprecipitation assays, cells were lysed with IP buffer (0.5% NP-40, 20 mM Tris-HCI (pH 7.5), 150 mM NaCl, 2 mM EDTA, 1.5 mM MgCl_2_) supplemented with protease inhibitor cocktail for 90 min on ice and centrifuged at 13,000 rpm for 10 min at 4°C. The supernatants were immunoprecipitated with the indicated antibody for 12 h at 4°C followed by incubation with protein A/G conjugated beads for 2 h. Beads were then washed with IP buffer and boiled in 2 x SDS-loading buffer. Protein samples were analyzed by immunoblotting.

### Subcellular Fractionation Assays

Purified mitochondria and subcellular fractionation assays were performed as previously reported (20). In brief, cells were harvested and homogenized in ice-cold mitochondrial isolation buffer (10 mM Tris-Cl (pH 7.4), 10 mM KCl, 1.5 mM MgCl_2_) supplemented with protease inhibitor cocktail (Sigma). Unbroken cells and nuclei were pelleted by centrifugation at 1,200 g for 10 min at 4°C. The pellet, containing mainly unbroken cells and nuclei, was washed twice in PBS and resuspended in nuclear buffer (150 mM NaCl, 1 mM KH_2_PO_4_, 5 mM MgCl_2_, 1 mM PMSF, 0.2 mM DTT, 0.3% Triton X-100, (pH 7.5)). Then, nuclear lysis buffer (50 mM Tris-Cl (pH 7.5), 500 mM NaCl, 10 mM β-mercaptoethanol, 1 mM EDTA, 10% glycerol, cocktail) was added to extract nuclear protein. The supernatants were centrifuged at 4°C for 30 min to pellet mitochondria. The supernatant resulting from the high-speed centrifugation (4,500 g), containing cytosoluble proteins, was centrifuged again at 8,000 g for 10 min at 4°C to avoid contamination from the mitochondrial fraction. The mitochondrial pellets were washed with mitochondrial isolation buffer and further centrifuged at 8,000 g for 20 min at 4°C. Lamin B1, tubulin, and TOM20 or COX4 were used as markers of nuclear, cytosolic, and mitochondrial proteins, respectively.

### Protease K Protection Assay

Proteinase K protection assays were performed as described (21, 22). Briefly, equal aliquots of freshly prepared mitochondria were treated with varying concentrations of proteinase K (Thermo) for 20 min on ice. Triton X-100 (1%) was eventually added to permeabilize the mitochondrial membranes and allow proteinase K to reach the mitochondrial matrix. Samples were then subjected to SDS-PAGE and immunoblotting. TOM70/MFN1, TIM23/COX4, and TFAM were used to analyze proteinase K-mediated degradation of proteins located in the outer mitochondrial membrane (OMM), the inner mitochondrial membrane (IMM), the mitochondrial matrix (MM), respectively.

### GST-Pull-Down Experiment

The cDNAs coding PGAM5 were cloned into the pET-22b (+) vector (Novagen) and cDNAs coding SND1 were cloned into the pGEX-4T-1 (GE) vector by the ClonExpress™ II One Step Cloning Kit (Vazyme). Proteins were produced in Escherichia coli (DE3). Purified GST fusion proteins and His-tagged proteins were used for pull-down experiments in pull-down buffer (50 mM Tris (pH 7.5), 150 mM NaCl, 5 mM DTT, 0.1% NP-40). After incubation, the beads were pelleted and washed with pull-down buffer followed by elution of proteins and analysis by western blot. The primer sequences used in these experiments are listed in online **Supplementary Table 1**.

### LC-MS and Data Analysis

The sample was prepared according to a preparation kit (Thermo: 90057), and LC-MS and data analysis were performed following the procedure described previously (23, 24). A Q-Exactive Plus mass spectrometer (Thermo) coupled to an EASY-nLC 1200 HPLC system (Thermo) with an anoelectrospray ion source and operated in data-dependent mode was used to analyze the samples. Raw LC-MS data were further analyzed using proteomics discovery software (Thermo Fisher Scientific, version 2.1) against the human UniProt database. A result was considered positive when the detected peptide constituted more than 5% of the total peptides in the protein. The immunoprecipitation mass spectrometry data of SND1-binding proteins relating to **Figure 3A** are available *via* ProteomeXchange with identifier PXD031036.

### Detection of Mitochondrial ROS

Mitochondrial ROS (mtROS) levels were detected using the MitoSOX Red fluorescence assay (Invitrogen, M36008). Cells were incubated with 5µM MitoSOX reagent in PBS buffer containing 5% FBS at 37°C for 30 min. The mitochondrial ROS levels were analyzed by flow cytometry (BD Biosciences) following the manufacturer’s instructions.

### Cell Viability Analysis

A total of 3 x 10^4^ cells suspended in 1 mL of medium were seeded in triplicate in 12-well plates. The cells in each well were trypsinized and counted every day after seeding. Data represent the means ± SD of three independent experiments.

### Animal Studies

All animal studies were conducted with approval from the Animal Research Ethics Committee of the South China University of Technology. Male BALB/c nude mice were purchased from SJA Laboratory Animal Company of China, and randomly assigned to experimental groups. For xenograft experiments, 7 × 10^6^ Hep3B cells were injected subcutaneously into 5-week-old male nude mice. Tumor volumes were measured every 3 days with a caliper and calculated using the equation volume = width (mm) x depth (mm) x length (mm) x 0.52.

### Clinical Human HCC Samples

Snap-frozen HCC tissues and the corresponding paracancerous tissues that were at least 2 cm away from the edge of the tumors were taken from 12 HCC patients by radical HCC resection at the First Affiliated Hospital of University of Science and Technology of China (Hefei, China). To use of these clinical materials for research purposes, prior written informed consent from the patients, as well as study approval from the Institutional Research Ethics Committee of Anhui Provincial Hospital was obtained. Total proteins were extracted from paired tissues and detected by immunoblotting.

### Statistical Analysis

The data are presented as the mean ± SD of at least three independent experiments. Student’s t-test was used to calculate *P* values. Statistical significance is indicated by * (*P* < 0.05) unless otherwise noted.

## Results

### SND1 Localizes to Mitochondria

**Figure 1:**
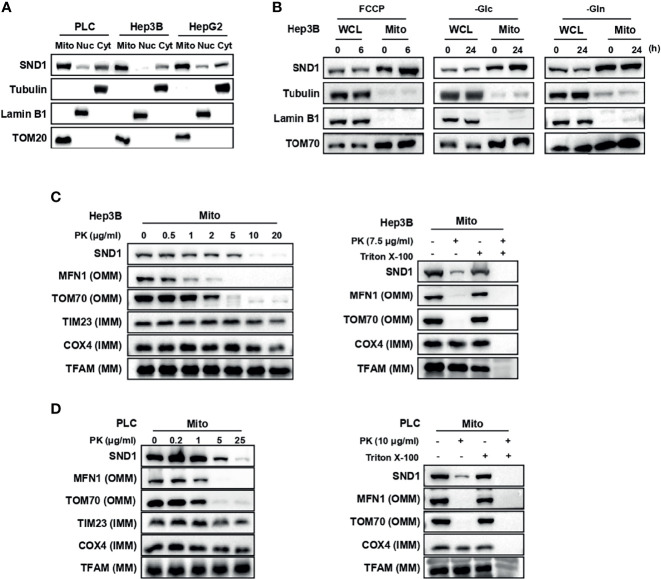
SND1 localizes to mitochondria. **(A)** Subcellular localization of endogenous SND1 in PLC, Hep3B, and HepG2 cells. Endogenous SND1 levels were determined by immunoblotting of fractions enriched in mitochondrial (Mito), nuclear (Nuc), and cytosolic (Cyt) proteins with an anti-SND1 antibody. Tubulin, Lamin B1, and translocase of outer mitochondrial membrane 20 (TOM20) were used as markers of cytosolic, nuclear, and mitochondrial proteins, respectively. **(B)** Immunoblotting analysis of endogenous SND1 protein levels in whole cell lysates (WCLs) and enriched mitochondrial fractions (Mitoes) in Hep3B cells treated with FCCP (left), glucose deprivation (middle), or glutamine deprivation (right) for the indicated hours, respectively. **(C, D)** Protease protection assays performed on purified mitochondria isolated from Hep3B cells **(C)** or PLC cells **(D)**. Enriched mitochondria were digested after incubation with proteinase K at the indicated concentrations for 20 min, and the localization of SND1 was analyzed by immunoblotting. The outer mitochondrial membrane (OMM) proteins MFN1 and TOM70, the inner mitochondrial membrane (IMM) proteins TIM23 and COX4, and the mitochondrial matrix (MM) protein TFAM were used as markers of OMM, IMM, and MM, respectively (left). Protease protection assays were performed in the presence of the permeabilizing agent 1% Triton X-100 (right).

Through the analysis of our IP-MS data, we unexpectedly found that SND1, a multifunctional regulator of gene expression in the nucleus, localizes to mitochondria (20) (**Supplementary Figure 1A**). MitoProt II (https://ihg.gsf.de/ihg/mitoprot.html) predicted that the probability of SND1 exportation to mitochondria was 82.23% (**Supplementary Figure 1B**), and MitoCarta3.0 also provided evidence to support the mitochondrial localization of SND1 in human tissues and mouse tissues (25) (**Supplementary Figure 1C**). To confirm this phenomenon, we evaluated the subcellular localization of SND1 in several liver cancer cell lines using isolated cellular compartments. Immunoblotting results showed that SND1 is obviously enriched in mitochondrial components in PLC, Hep3B, and HepG2 cell lines (**Figure 1A**).

To explore the role of the tumor microenvironment on SND1 sublocalization, FCCP (carbonyl cyanide p-(trifluoromethoxy) phenylhydrazone), a proton ionophore widely employed to study the role of mitochondria in cell function, and nutrient stresses including glucose deprivation and glutamine deprivation were used to simulate the tumor microenvironment. Immunoblotting results showed that there was no significant change in the total SND1 protein levels after indicated treatment, but the accumulation of SND1 in mitochondria was further enhanced under FCCP treatment or glucose deprivation conditions, not under glutamine deprivation conditions (**Figure 1B**). These results suggest that SND1 plays a role closely related to mitochondria within the tumor microenvironment.

### SND1 Localizes in the Outer and Inner Membranes of Mitochondria

As shown in **Supplementary Figure 1C**, the mitochondrial sublocalization of SND1 is unknown compared to other known mitochondrial proteins. Therefore, we performed a proteinase K protection assay to study the precise mitochondrial sublocalization of endogenous SND1 in purified mitochondria of Hep3B cells. As shown in **Figure 1C**, after treatment with proteinase K, the outer mitochondrial membrane (OMM) proteins MFN1 and TOM70 disappeared, and the inner membrane protein COX4 was digested gradually with increasing proteinase K concentration (**Figure 1C**, left), whereas the mitochondrial matrix protein TFAM was protected from proteolysis by proteinase K (**Figure 1C**, left), unless Triton X-100 was added (**Figure 1C**, right). SND1 was not protected by proteinase K, but the digestion rate of SND1 was between that of OMM protein and IMM protein (**Figure 1C**). Similar results were observed in PLC cells (**Figure 1D**). Taken together, our data indicate that SND1 mainly localizes in the outer membrane of mitochondria, but it also has a slight localization in the inner membrane.

### TOM70 Is Required for the Import of SND1 Into Mitochondria

**Figure 2:**
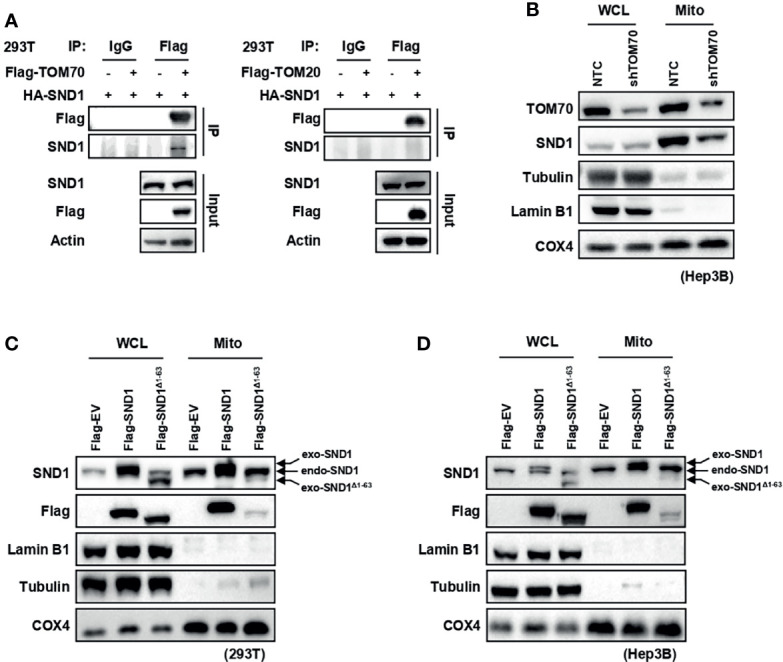
TOM70 is required for the import of SND1 into mitochondria. **(A)** Co-Immunoprecipitation assay of the protein interaction between SND1 and TOM70 (left) or TOM20 (right). HEK293T (293T) cells were transfected with HA-SND1 plasmids alone or together with Flag-TOM70 or Flag-TOM20 plasmids. Cell lysates were immunoprecipitated with anti-Flag antibody, followed by immunoblotting analysis with antibodies against Flag and SND1. Actin served as loading control. **(B)** Immunoblotting analysis of SND1 protein levels in the WCL and mitochondrial fraction of Hep3B cells stably expressing shTOM70. Tubulin/Lamin B1 and COX4 served as loading controls for the WCL and mitochondrial fraction, respectively. **(C, D)** Immunoblotting analysis of SND1 protein levels in the WCL and mitochondrial fraction of 293T **(C)** or Hep3B **(D)** cells expressing Flag-EV, Flag-SND1, or Flag-SND1^Δ1-63^. Tubulin/Lamin B1 and COX4 served as loading controls for the WCL and mitochondrial fraction, respectively.

Mitochondrial outer membrane proteins TOM20 and TOM70, known as import receptors, form the translocase of the outer membrane (TOM complex) with TOM22 and TOM40 to promote the import of mitochondrial proteins from the cytoplasm into the mitochondria (26, 27). Through Co-Immunoprecipitation (Co-IP) experiments, we found that SND1 overexpressed in 293T cells specifically bound to the TOM70 protein, but not the TOM20 protein (**Figure 2A**). More importantly, the translocation of SND1 into mitochondria was markedly decreased when TOM70 was knocked down by shRNA in Hep3B cells (**Figure 2B**). In summary, our results indicate that the import receptor TOM70 is responsible for the mitochondrial localization of SND1. Further bioinformatics analysis of the SND1 protein sequence with the MitoProt II website revealed that the first 63 amino acids of SND1 were its potential mitochondrial targeting sequence (MTS) (**Supplementary Figure 1B**). Then we overexpressed wild-type SND1 and the SND1^Δ1-63^ mutant in 293T and Hep3B cells, respectively, to study the role of the first 63 amino acids on the localization of SND1. Immunoblotting results showed that Flag-tagged wild-type SND1 was enriched in mitochondria, but deletion of the first 1-63 amino acid of SND1 inhibited the mitochondrial localization of Flag-tagged SND1^Δ1-63^, while the enrichment of endogenous SND1 in mitochondria was not affected (**Figures 2C, D**). Collectively, our results indicate that the N-terminal amino acids 1-63 of SND1 act as an MTS to help SND1 be recognized by Tom70, which further promotes SND1 entry into mitochondria.

### SND1 Binds to PGAM5 in Mitochondria

**Figure 3:**
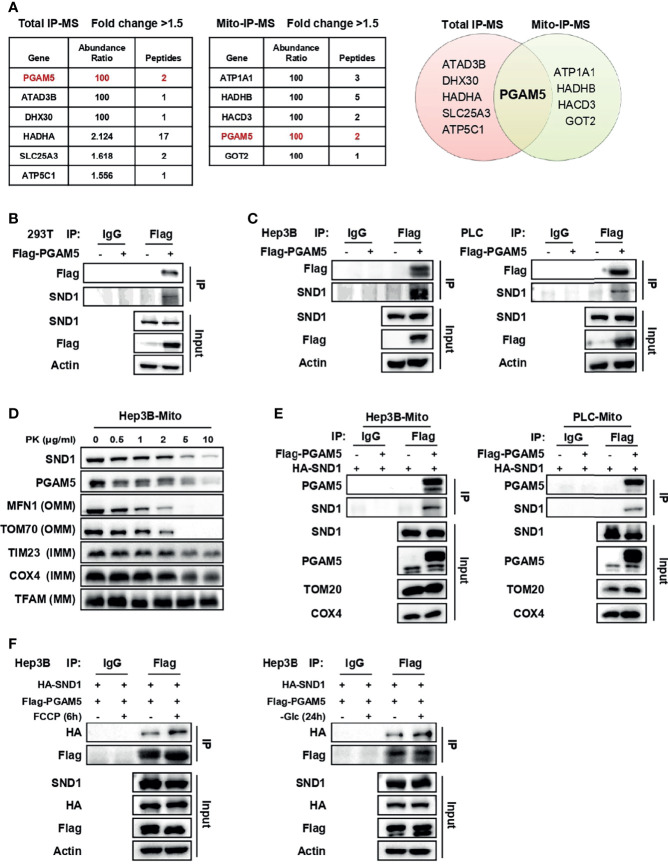
SND1 binds to PGAM5 in mitochondria. **(A)** Proteins bound by SND1 with a fold change > 1.5 compared to the EV control group in total IP-MS or mitochondrial IP-MS results (left). Venn diagram showing the overlap of proteins bound by SND1 from total IP-MS and mitochondrial IP-MS results (right). **(B)** 293T cells were transfected with Flag-EV or Flag-PGAM5 plasmids. Cell lysates were immunoprecipitated with anti-Flag antibody, followed by immunoblotting analysis with antibodies against Flag and SND1. Actin served as loading control. **(C)** Hep3B cells (left) or PLC cells (right) stably expressing Flag-PGAM5 were harvested and subjected to Co-IP analysis. Cell lysates were immunoprecipitated with anti-Flag antibody, followed by immunoblotting analysis with antibodies against Flag and SND1. Actin served as loading control. **(D)** Protease protection assays performed on purified mitochondria isolated from Hep3B cells. Enriched mitochondria were digested after incubation with proteinase K at the indicated concentrations and the localization of PGAM5 was analyzed by immunoblotting. MFN1/TOM70, TIM23/COX4, and TFAM were used as markers of OMM, IMM, and MM, respectively. **(E)** Hep3B cells (left) or PLC cells (right) stably expressing HA-SND1 were further infected with viruses expressing Flag-EV or Flag-PGAM5, and then Co-IP assays were performed using an anti-Flag antibody in enriched mitochondrial fractions, followed by immunoblotting analysis with antibodies against PGAM5 and SND1. TOM20 and COX4 served as loading control. **(F)** Hep3B cells stably expressing HA-SND1 were further infected with viruses expressing Flag-PGAM5, and then treated with FCCP for 6 h (left) or glucose-free medium (right) for 24 h. Cells were harvested and cell lysates were subjected to Co-IP analysis using an anti-Flag antibody, followed by immunoblotting analysis with antibodies against HA, Flag, and SND1. Actin served as loading control.

We then asked whether the mitochondrial localization of SND1 affects the function of mitochondria and performed immunoprecipitation-mass spectrometry (IP-MS) to determine which mitochondrial proteins bound to SND1. In PLC cells overexpressing Flag-tagged SND1, immunoprecipitation (IP) assays were performed in total cell lysates or purified mitochondrial lysates with anti-Flag antibody (**Supplementary Figures 2A, B**). According to the analysis of the binding proteins of SND1, PGAM5 was the protein immunoprecipitated by SND1 in both total IP-MS and mito-IP-MS (**Figure 3A**). Co-IP experiments in 293T cells revealed that PGAM5 binds to exogenous HA-tagged SND1 or endogenous SND1 (**Supplementary Figure 2C** and **Figure 3B**). We further overexpressed Flag-tagged PGAM5 in both Hep3B and PLC cells and found that the endogenous SND1 could bind to immunoprecipitated Flag-tagged PGAM5 (**Figure 3C**). Interestingly, a proteinase K protection assay showed that PGAM5 was located in both the outer and inner membranes of mitochondria (**Figure 3D**), which was very consistent with the localization trend of SND1 (**Figures 1C, D**).

To explore whether SND1 bound to PGAM5 in mitochondria, we overexpressed SND1 and PGAM5 in Hep3B and PLC cells. Purified mitochondria were subjected to IP analysis with an anti-Flag antibody, and the results showed that SND1 indeed bound to PGAM5 in mitochondria (**Figure 3E**). More importantly, the binding ability of SND1 and PGAM5 was further enhanced upon FCCP or glucose deprivation treatment, suggesting that the binding of SND1 and PGAM5 plays an important role within the tumor microenvironment (**Figure 3F**).

### The C-Termini of Both SND1 and PGAM5 Mediate the Binding of SND1 and PGAM5

**Figure 4:**
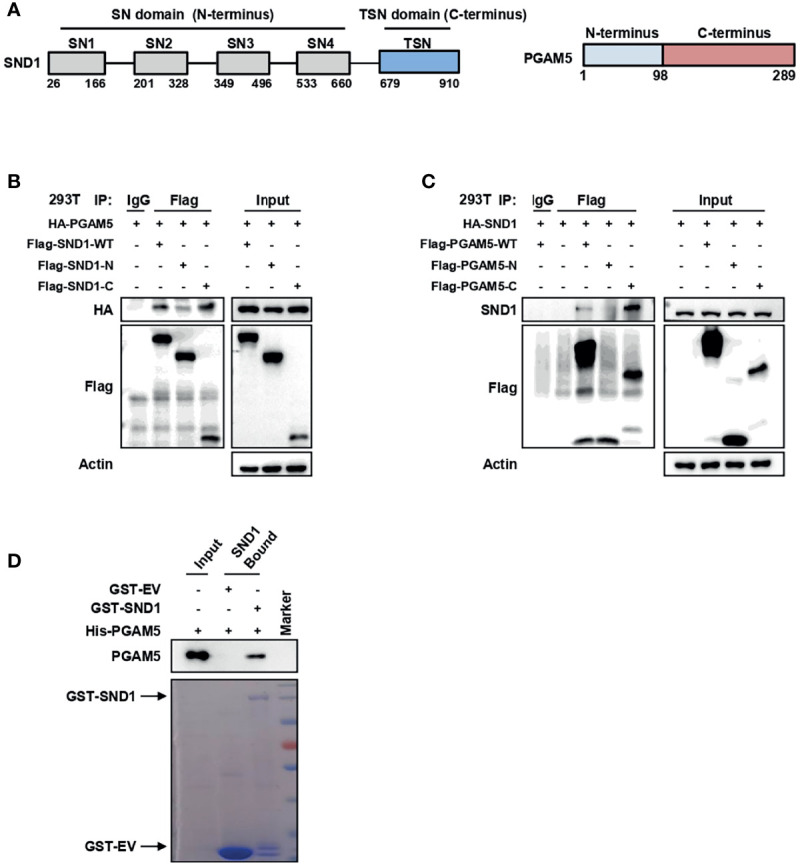
The C-termini of both SND1 and PGAM5 mediate the binding of SND1 and PGAM5. **(A)** The diagrams show the structures of SND1 and PGAM5. **(B)** 293T cells were transfected with HA-PGAM5 plasmids alone or together with Flag-tagged plasmids expressing full-length SND1 (WT), its SN domains (N-terminus) or the TSN domain (C-terminus). Cell lysates were immunoprecipitated with anti-Flag antibody or IgG, followed by immunoblotting analysis with antibodies against HA and Flag. Actin served as loading control. **(C)** 293T cells were transfected with HA-SND1 plasmids alone or together with Flag-tagged plasmids expressing full-length PGAM5 (WT), the N-terminus, or the C-terminus. Cell lysates were immunoprecipitated with anti-Flag antibody or IgG, followed by immunoblotting analysis with antibodies against SND1 and Flag. Actin served as loading control. **(D)** GST pull-down of His-PGAM5 by GST-SND1 using protein purified from *E. coli*, followed by immunoblotting analysis with anti-PGAM5 and anti-GST antibodies.

SND1 consists of a tandem repeat of four staphylococcal nuclease (SN) domains at the N-terminus followed by a Tudor domain and a truncated SN domain (TSN domain) at the C-terminus (28) (**Figure 4A**, left panel). Using vectors expressing the N-terminus and C-terminus of SND1, we further demonstrated that PGAM5 mainly bound to the C-terminus of SND1 (**Figure 4B**). To identify the residues on the PGAM5 protein responsible for its binding to SND1, we constructed vectors expressing the WT, N-terminus (1-98), or C-terminus (99-289) of the PGAM5 protein (**Figure 4A**, right panel). Co-IP results showed that the C-terminus, but not the N-terminus, bound to SND1 (**Figure 4C**). In addition, a pull-down assay using the purified recombinant proteins demonstrated the direct interaction between SND1 and PGAM5 (**Figure 4D**).

### SND1 Promotes Mitophagy Through PGAM5

**Figure 5:**
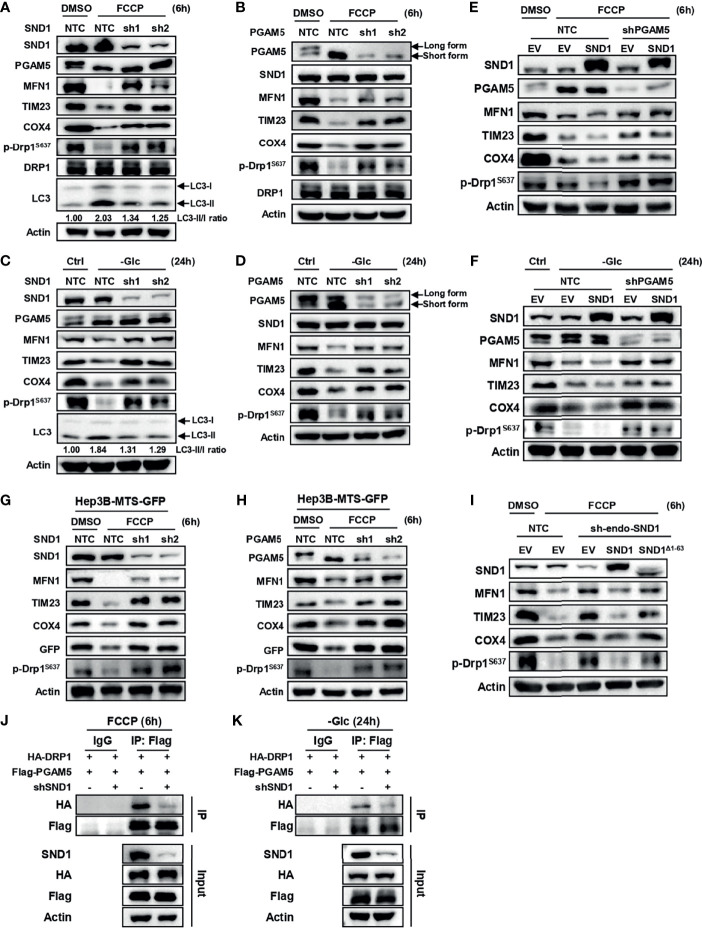
SND1 promotes mitophagy through PGAM5. **(A, B)** Hep3B cells stably expressing shSND1 **(A)** or shPGAM5 **(B)** were treated with 10 μM FCCP for 6 h. Samples were collected for immunoblotting to analyze the expression of SND1, PGAM5, MFN1, TIM23, COX4, p-DRP1^S637^, and DRP1. Actin served as loading control. LC3 protein levels were detected in Hep3B-shSND1 cells treated with FCCP. **(C, D)** Hep3B cells stably expressing shSND1 **(C)** or shPGAM5 **(D)** were treated with glucose-free medium for 24 h. Samples were collected for immunoblotting to analyze the expression of SND1, PGAM5, MFN1, TIM23, COX4, and p-DRP1^S637^. Actin served as loading control. LC3 protein levels were detected in Hep3B-shSND1 cells treated with glucose-free medium. **(E, F)** Hep3B cells stably overexpressing SND1 with further PGAM5 knockdown by shRNAs were treated with FCCP for 6 h **(E)** or cultured with glucose-free medium for 24 h (**F**), followed by immunoblotting analysis with antibodies against SND1, PGAM5, MFN1, TIM23, COX4, and p-DRP1^S637^. Actin served as loading control. **(G, H)** Hep3B cells stably overexpressing MTS-GFP with further SND1 knockdown **(G)** or PGAM5 knockdown **(H)** were cultured with FCCP for 6 h, followed by immunoblotting analysis with antibodies against SND1, PGAM5, MFN1, TIM23, COX4, GFP, and p-DRP1^S637^. Actin served as loading control. **(I)** Hep3B cells with endogenous SND1 knockdown were further infected with viruses expressing Flag-EV, Flag-SND1, or Flag-SND1^Δ1-63^. These cells were treated with FCCP for 6 h followed by immunoblotting analysis with antibodies against SND1, MFN1, TIM23, COX4, and p-DRP1^S637^. Actin served as loading control. **(J, K)** Hep3B cells were infected virus expressing HA-DRP1 together with Flag-PGAM5 plasmids, and the binding activity of PGAM5 and DRP1 under FCCP treatment **(J)** or glucose deprivation conditions **(K)** was determined when we further knocked down SND1. Cell lysates were immunoprecipitated with anti-Flag antibody or IgG, followed by immunoblotting analysis with antibodies against HA, Flag, and SND1. Actin served as loading control.

Because FCCP is a potent inducer of mitophagy and PGAM5 was reported to participate in mitophagy (29–32), we investigated whether SND1 regulates mitophagy through PGAM5. In Hep3B cells with SND1 knockdown further treated with FCCP for 6 h, we found that, as a disruptor of mitochondrial membrane potential, FCCP-induced degradation of mitochondrial proteins such as MFN1, TIM23, and COX4 due to mitophagy (22, 30), and this phenomenon was inhibited by SND1 knockdown (**Figure 5A**). Similar results were observed when we knocked down PGAM5 in Hep3B cells that were further treated with FCCP for 6 h (**Figure 5B**). FCCP induced the enrichment of short-form PGAM5 (PGAM5S) by splicing from long-form PGAM5 (PGAM5L), which is consistent with a previous report (30). PGAM5 regulates mitophagy through dephosphorylation of DRP1, a substrate of PGAM5 (31). Our result showed that FCCP-induced dephosphorylation of DRP1 was also partially inhibited when we knocked down SND1 or PGAM5 (**Figures 5A, B**). Glucose deprivation-induced mitophagy, such as the degradation of MFN1, TIM23, and COX4 and dephosphorylation of DRP1^S637^, was also repressed when we knocked down SND1 or PGAM5 (**Figures 5C, D**). In addition, upon FCCP or glucose-free medium treatment, the increased LC3II/I ratio and accumulated mitoROS levels, two other indicators of mitophagy, were significantly suppressed when knockdown of SND1 (**Figures 5A, C** and **Supplementary Figure 3**). Importantly, SND1 promotes mitophagy by further enhancing the degradation of MFN1, TIM23, COX4, and dephosphorylation of DRP1^S637^ compared to the FCCP-treated EV groups, but this promoting effect disappeared when we further knocked down PGAM5 in SND1-overexpressing Hep3B cells (**Figure 5E**). Similar results were observed under glucose deprivation conditions (**Figure 5F**). These results suggest that SND1-promoted mitophagy is mediated by PGAM5.

To further illustrate this phenomenon, we overexpressed mitochondria-targeted green fluorescent protein (MTS-GFP) in Hep3B cells and explored the role of SND1 and PGAM5 in mitophagy by examining the degradation of MTS-GFP. The results showed that FCCP or glucose deprivation treatment significantly promoted the degradation of MTS-GFP, which was rescued when we further knocked down SND1 or PGAM5 (**Figures 5G, H**).

### SND1-Induced Mitophagy Depends on the Mitochondrial Targeting Sequence

To evaluate whether SND1-induced mitophagy is dependent on its mitochondrial localization, we overexpressed wild-type SND1 and SND1^Δ1-63^ mutant in endogenous SND1-knockdown Hep3B cells and treated these cells with FCCP for 6 h. Immunoblotting results showed that knockdown of SND1 attenuated FCCP-induced mitophagy, which was recovered by further overexpression of wild-type SND1, but not the SND1^Δ1-63^ mutant (**Figure 5I**). This result indicates that mitochondrial localization of SND1 mediated by the first 63 amino acids is required for it to promote mitophagy. DRP1 dephosphorylation mediated by PGAM5 binding to DRP1 is another indicator of PGAM5-mediated mitophagy (33–35). Our Co-IP results showed that knockdown of SND1 significantly reduced the binding ability of PGAM5 to DRP1 induced by FCCP or glucose deprivation (**Figures 5J, K**), suggesting that SND1 is required for PGAM5-mediated DRP1 dephosphorylation and subsequent mitophagy.

### The Effects of SND1 on Promoting Tumor Growth Depends on PGAM5 and Its Mitochondrial Localization

**Figure 6:**
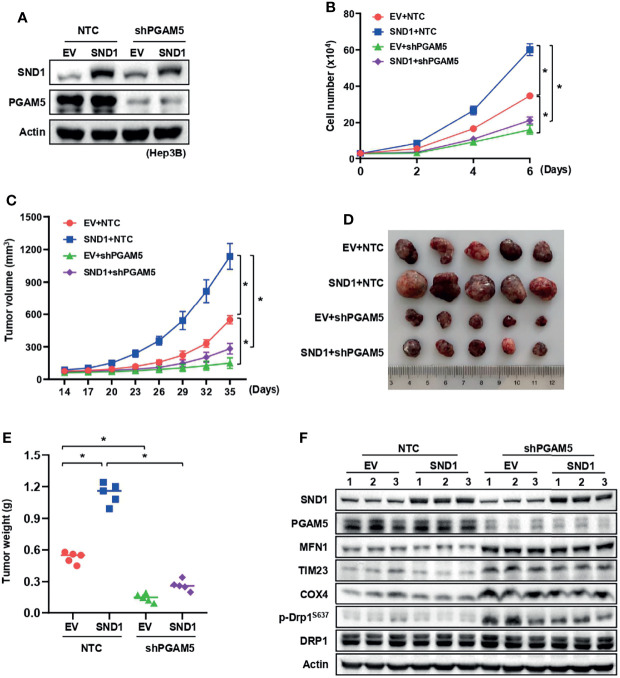
The promotion effect of SND1 on cell proliferation and tumor growth depends on PGAM5. **(A, B)** Growth curves were measured in SND1-overexpressing Hep3B cells expressing shRNA against PGAM5. Data are presented as the mean ± SD of three independent experiments. *P < 0.05 comparing the indicated samples. **(C–E)** Equal numbers of Hep3B cells mentioned in panel **(A)** were injected subcutaneously into the flanks of BALB/c nude mice (n = 5 mice in each group). Tumor sizes were measured every 3 days using calipers **(C)**. Photographs show xenografts **(D)**, and tumor weights **(E)** were determined at the end of the experiment (Day 35). Data are presented as the mean ± SD, *P < 0.05 comparing the indicated groups. **(F)** Protein levels of SND1, PGAM5, MFN1, TIM23, COX4, p-DRP1^S637^, and DRP1 in tumor lysates of panel **D** were measured by immunoblotting analysis. Actin served as loading control.

**Figure 7:**
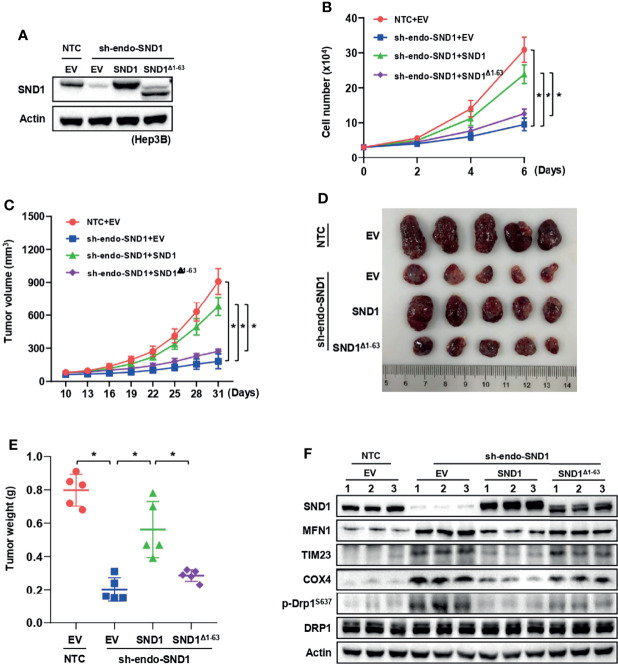
Mitochondrial localization is required for SND1-mediated cell proliferation and tumor growth. **(A, B)** Growth curves of Hep3B cells with endogenous SND1 knockdown that were expressing Flag-EV, Flag-SND1, or Flag-SND1^Δ1-63^ were determined by trypan blue counting. Data are presented as the mean ± SD of three independent experiments. *P < 0.05 comparing the indicated samples. **(C–E)** Equal numbers of Hep3B cells mentioned in panel **(A)** were injected subcutaneously into the flanks of BALB/c nude mice (n = 5 mice in each group). Tumor sizes were measured every 3 days using calipers **(C)**. Photographs show xenografts **(D)**, and tumor weights **(E)** were determined at the end of the experiment (Day 31). Data are presented as the mean ± SD, *P < 0.05 comparing the indicated groups. **(F)** Protein levels of SND1, MFN1, TIM23, COX4, p-DRP1^S637^, and DRP1 in tumor lysates of panel **(D)** were measured by immunoblotting analysis. Actin served as loading control.

To address whether PGAM5-mediated mitophagy and mitochondrial localization of SND1 are necessary for SND1 regulation of cell proliferation and tumor growth, we performed cell growth assays and xenograft experiments using Hep3B cells. As previously reported (36, 37), stable overexpression of SND1 promoted cell proliferation and tumor growth in Hep3B cells compared with the control empty vector group (**Figures 6A–C**). However, when PGAM5 was knocked down, SND1-enhanced cell proliferation, tumor growth and weight of Hep3B xenografts were markedly reduced (**Figures 6B–E**), suggesting that PGAM5 was critical for SND1-regulated cell growth *in vitro* and tumor growth *in vivo*. Immunoblotting using tumor tissue lysates confirmed the overexpression of SND1 and the knockdown of PGAM5 by shRNAs in Hep3B xenografts (**Figure 6F**). Detection of MFN1, TIM23, COX4, and phosphorylation of DRP1^S637^ also revealed that SND1-induced mitophagy was repressed when PGAM5 was knocked down (**Figure 6F**). Additionally, we further examined the effect of mito-SND1 on cell proliferation and tumor growth. Cell growth assays showed that overexpression of wild-type SND1, but not the SND1^Δ1-63^ mutant, dramatically rescued endogenous SND1 knockdown-induced cell growth defects (**Figures 7A, B**). Similar results were observed in tumor growth in the mouse model (**Figures 7C–E**). Using tumor tissue lysates, we further confirmed the knockdown of endogenous SND1 and overexpression of wild-type SND1 and the SND1^Δ1-63^ mutant (**Figure 7F**). Importantly, we found that overexpression of wild-type SND1, but not the SND1^Δ1-63^ mutant, dramatically rescued the endogenous SND1 knockdown-repressed mitophagy by detecting the protein levels of MFN1, TIM23, COX4 and phosphorylation of DRP1^S637^ (**Figure 7F**). These results demonstrate that PGAM5 and mitochondrial localization of SND1 are required for the promotion effect of SND1 on mitophagy, cell proliferation, and tumor growth.

### SND1 and PGAM5 Are Potential Prognostic Markers for HCC Patients

**Figure 8:**
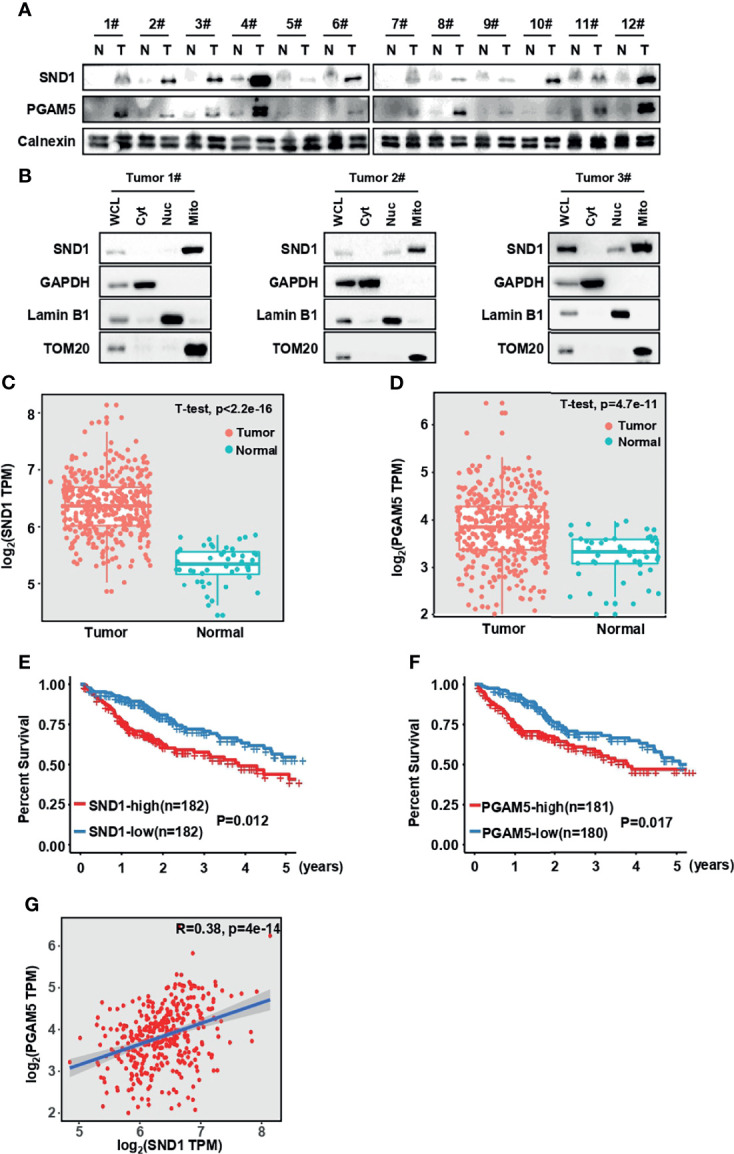
SND1 and PGAM5 are potential prognostic markers for HCC patients. **(A)** Immunoblotting analysis of SND1 and PGAM5 protein levels in 12 pairs of clinically matched adjacent noncancerous liver tissues (Normal) and human liver cancer tissues (Tumor). Calnexin served as loading control. **(B)** Immunoblotting analysis of SND1 protein levels in Cyt, Nuc, and Mito components purified from tumor tissues of HCC patients. GAPDH, Lamin B1, and TOM20 were used as markers of cytosolic, nuclear, and mitochondrial proteins, respectively. **(C, D)** mRNA levels of SND1 **(C)** and PGAM5 **(D)** were determined in adjacent noncancerous liver tissues (Normal) and liver cancer tissues (Tumor) from The Cancer Genome Atlas (TCGA) database (http://gepia.cancer-pku.cn/). **(E, F)** Kaplan-Meier analysis of overall survival with log-rank tests for low versus high expression of SND1 or PGAM5 genes in HCC patients (http://gepia.cancer-pku.cn/). **(G)** SND1 is positively correlated with the expression of PGAM5 in liver cancer tissues based on TCGA cohort. The line indicates linear regression, and Spearman’s rank correlation coefficients (R) and the corresponding *p* value are indicated.

To further investigate the pathological significance of our findings, we examined SND1 and PGAM5 expression in 12 paired clinical human HCC lesions and adjacent noncancerous tissue samples. The results showed that the SND1 and PGAM5 levels were significantly increased in the HCC lesions compared to the adjacent noncancerous tissue (**Figure 8A**). Using isolated cellular compartments from tumor tissues of liver cancer patients, we further found that SND1 was indeed enriched in mitochondria (**Figure 8B**). The mRNA levels of SND1 and PGAM5 were all enriched in HCC lesions compared with normal liver tissues through analysis of TCGA database (**Figures 8C, D**) (http://gepia.cancer-pku.cn/) (38). Additionally, the association of SND1 and PGAM5 with liver cancer patients was further determined by prognostic analyses. The Kaplan-Meier test indicated that patients with high expression of SND1 and PGAM5 were significantly associated with poor prognosis (**Figures 8E, F**). Finally, Person correlation analysis revealed that SND1 is significantly and positively coexpressed with PGAM5 according to the liver cancer database of TCGA, and SND1 thus positively regulates mitophagy through PGAM5 (**Figure 8G**).

## Discussion

**Figure 9:**
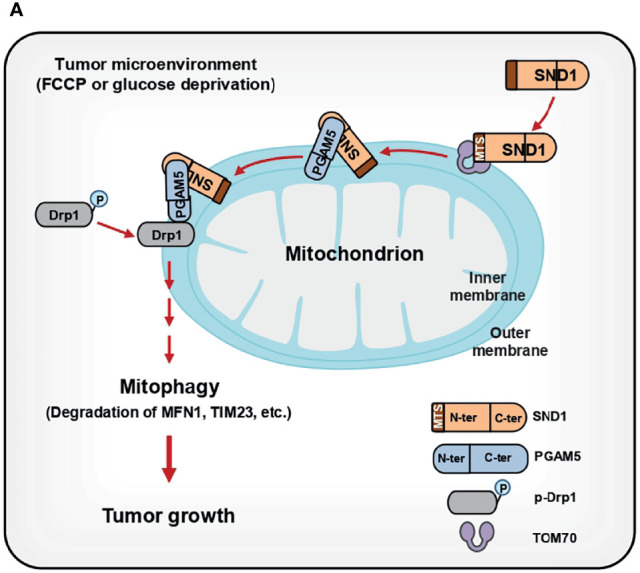
Summary of SND1-mediated mitophagy through PGAM5 during tumor progression. **(A)** Summary: SND1, as an evolutionarily conserved protein, is unexpectedly enriched in the outer and inner membranes of mitochondria through its MTS with the assistance of TOM70. In the mitochondria, SND1 binds to PGAM5 through its C-terminus and this interaction is crucial for SND1-mediated mitophagy, such as the degradation of MFN1, TIM23, and COX4, and dephosphorylation of p-DRP1^S637^. Finally, SND1-induced advantages of cell proliferation and tumor growth depend on PGAM5-mediated mitophagy and its mitochondrial localization.

As a multifunctional regulator of gene expression, SND1 regulates cellular activity mainly through its nuclear functions. Recently, Aviram et al. revealed that SND proteins facilitate the endoplasmic reticulum (ER) targeting of substrates containing targeting motifs in yeast (39), and Wang et al. further identified SND1 as an ER membrane-associated protein facilitating immune evasion (40). However, it remains unknown whether SND1 is located in mitochondria and performs related functions. Our MS results showed that SND1 is enriched in the mitochondrial fraction (20), and this phenomenon was confirmed by subcellular fractionation. By performing cell growth and xenograft assays, we find that SND1-induced mitophagy promotes cell proliferation and tumor progression, depending on PGAM5 and its mitochondrial localization (**Figure 9**).

Increased dysregulation of mitochondria is recognized to be a major cause of many metabolic diseases, neurodegenerative disorders, aging, and cancer. Selective removal of damaged mitochondria by mitophagy is used to maintain quality control in physiological and pathological contexts, but the roles of mitophagy in tumor progression are controversial due to the different levels of mitophagy or different stages of cancers (19). In this study, we found that SND1 promotes liver cancer through PGAM5-mediated Drp1^S637^ dephosphorylation and mitophagy (**Figures 5**, **6**). Drp1 is a mitochondrial fission regulator and essential for mitochondrial division. Phosphorylation of Drp1 is known to regulate mitochondrial dynamics. Suppression of Drp1-mediated mitochondrial fission and mitophagy promotes cell apoptosis under hypoxic conditions (41). Additionally, many studies have shown that PGAM5 and DRP1 can regulate apoptosis, necrosis, and mitophagy, thus acting as hubs for multiple models of cell death (33, 35). Our results showed that SND1 promotes the interaction between PGAM5 and DRP1 by binding to PGAM5, thereby promoting mitophagy (**Figures 5J, K**). This evidence suggests that PGAM5/DRP1-mediated mitochondrial dynamics may be involved in SND1-mediated mitophagy under FCCP stimulation or nutrient stress conditions.

In addition to regulating transcription as a cotranscriptional factor, SND1 also acts as an RNA binding protein to regulate mRNA splicing and mRNA stability. SND1 is known to upregulate angiotensin II type 1 receptor (AT1R) expression by binding to AT1R’s 3’ UTR to promote its mRNA stability (42). Cappellari et al. found that SND1 regulates CD44 exon v5 splicing by forming a complex with SAM68 (43). More recently, SND1 has been found to interact with MTDH to form complexes (13, 14, 16). This complex binds and disrupts the stability of *Tap1/2* mRNAs, which encode key components of the antigen-presentation machinery, thereby inhibiting tumor antigen presentation and T-cell activation, leading to immune evasion in triple-negative breast cancer (14). Mitochondria are semiautonomous organelles because they possess their own DNA that encodes mitochondrial RNAs (mt-RNAs), including mRNA, rRNA, and tRNAs. Given the importance of post-transcriptional regulation of RNA stability, splicing, and decay in the regulation of gene expression, whether mito-SND1 is involved in the maintenance of mitochondrial homeostasis through regulation mt-RNA processing is worth further exploration. Mitochondria are dynamic organelles that act as the powerhouse of cells, and they have thus received considerable attention related to the role of metabolic reprogramming in tumorigenesis. SND1 is known to control adipogenesis and glycerophospholipid metabolism as a transcriptional coactivator (44, 45). Our mito-IP-MS results showed that SND1 potentially binds to HADHB, HACD3, and GOT2 (**Figure 3A**). These three enzymes participate in lipid metabolism and amino acid metabolism. However, it is not clear whether SND1 is involved in metabolic reprogramming by binding to these mitochondrial metabolic enzymes.

In conclusion, we revealed the previously unknown localization of SND1 to mitochondria, which regulates mitophagy, cell growth, and tumor progression. Although the exact functions of SND1 in prokaryotes and eukaryotes have not been fully explained, our findings place SND1 within mitochondria and suggest that SND1 serves as a modulator of mitophagy by entering mitochondria and binding to PGAM5 in response to external stimulation and nutrient stress in human liver cancers. We conclude that SND1 and PGAM5 may be potential therapeutic targets in HCC patients.

## Data Availability Statement

The datasets presented in this study can be found in online repositories. The names of the repository/repositories and accession number(s) can be found in the article/**Supplementary Material**.

## Ethics Statement

The studies involving human participants were reviewed and approved by the First Affiliated Hospital of University of Science and Technology of China. The patients/participants provided their written informed consent to participate in this study. The animal study was reviewed and approved by the Animal Research Ethics Committee of the South China University of Technology.

## Author Contributions

SLia, CZ, CS, HW, YY, XG, LC, MY, and SLi performed the experiments, SLia, PG, and LS wrote the manuscript. SS performed the bioinformatics analysis. SLia, CZ, and HW performed the in vivo experiment. PG and LS designed and reviewed the study. All authors contributed to the article and approved the submitted version.

## Funding

This work is supported in part by National Key R&D Program of China (2018YFA0800300), National Natural Science Foundation of China (81874060), and the Program for Guangdong Introducing Innovative and Entrepreneurial Teams (2017ZT07S054).

## Conflict of Interest

The authors declare that the research was conducted in the absence of any commercial or financial relationships that could be construed as a potential conflict of interest.

## Publisher’s Note

All claims expressed in this article are solely those of the authors and do not necessarily represent those of their affiliated organizations, or those of the publisher, the editors and the reviewers. Any product that may be evaluated in this article, or claim that may be made by its manufacturer, is not guaranteed or endorsed by the publisher.

## Supplementary Material

Supplementary Figure 1 SND1 localizes to mitochondria. **(A)** SND1 is a potential mitochondrial protein from our previous IP-MS result (Li et al. Nature Metabolism, 2020). **(B)** MitoProt II (https://ihg.gsf.de/ihg/mitoprot.html) predicts the probability of SND1 exportation to mitochondria and the mitochondrial targeting sequence (MTS) of SND1. **(C)** MitoCarta3.0 provides evidence supporting the mitochondrial localization of SND1 in human tissues and mouse tissues.

Supplementary Figure 2 SND1 binds to PGAM5. **(A, B)** In PLC cells overexpressing with Flag-tagged SND1, immunoprecipitation assays were performed in total cell lysates **(A)** or purified mitochondrial lysates **(B)** with anti-Flag antibody, followed by immunoblotting analysis with antibodies against Flag and SND1. Actin served as loading control. **(C)** 293T cells expressing HA-SND1 were further transfected with Flag-EV or Flag-PGAM5 plasmids. Cell lysates were immunoprecipitated with anti-Flag antibody, followed by immunoblotting analysis with antibodies against Flag and SND1. Actin served as loading control.

Supplementary Figure 3 Knockdown of SND1 suppresses mtROS accumulation induced by FCCP treatment or glucose-free medium. **(A)** Hep3B cells stably expressing shSND1 were treated with 10 μM FCCP for 6 h. Samples were collected and the mitochondrial ROS levels were analyzed by flow cytometry. **P* < 0.05 comparing with the indicated groups. **(B)** Hep3B cells stably expressing shSND1 were treated with glucose-free medium for 24 h. Samples were collected and the mitochondrial ROS levels were analyzed by flow cytometry. * *P* < 0.05 comparing with the indicated groups.
