# Supplementary figures and images for: Mitochondrion-Localized SND1 Promotes Mitophagy and Liver Cancer Progression Through PGAM5
Source: Front Oncol. 2022 Mar 31;12:857968. doi: 10.3389/fonc.2022.857968 (PMC9008731; doi:10.3389/fonc.2022.857968)

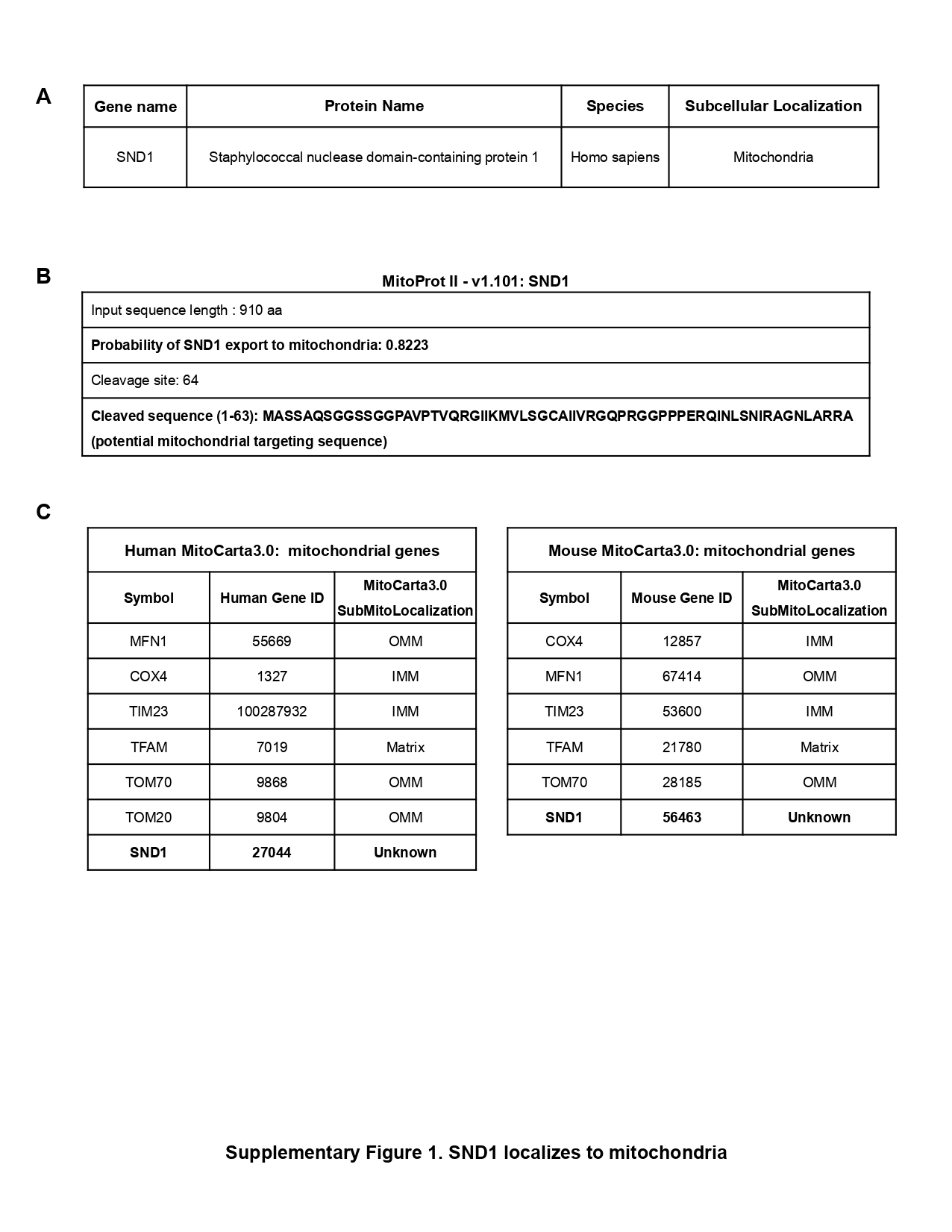

Supplement: Supplementary Figure 1 — SND1 localizes to mitochondria. (A) SND1 is a potential mitochondrial protein from our previous IP-MS result (Li et al. Nature Metabolism, 2020). (B) MitoProt II (https://ihg.gsf.de/ihg/mitoprot.html) predicts the probability of SND1 exportation to mitochondria and the mitochondrial targeting sequence (MTS) of SND1. (C) MitoCarta3.0 provides evidence supporting the mitochondrial localization of SND1 in human tissues and mouse tissues. [file Image_1.jpg]

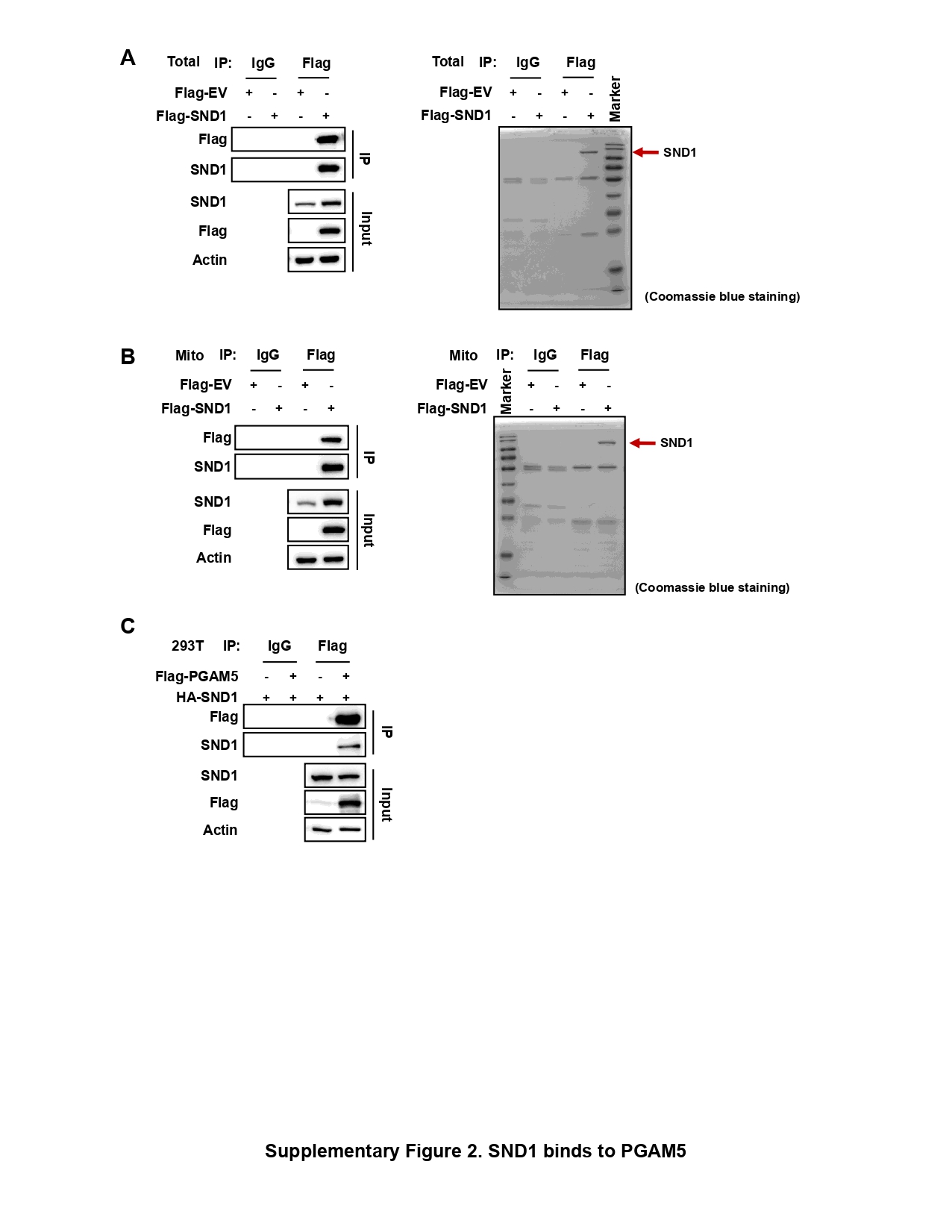

Supplement: Supplementary Figure 2 — SND1 binds to PGAM5. (A, B) In PLC cells overexpressing with Flag-tagged SND1, immunoprecipitation assays were performed in total cell lysates (A) or purified mitochondrial lysates (B) with anti-Flag antibody, followed by immunoblotting analysis with antibodies against Flag and SND1. Actin served as loading control. (C) 293T cells expressing HA-SND1 were further transfected with Flag-EV or Flag-PGAM5 plasmids. Cell lysates were immunoprecipitated with anti-Flag antibody, followed by immunoblotting analysis with antibodies against Flag and SND1. Actin served as loading control. [file Image_2.jpg]

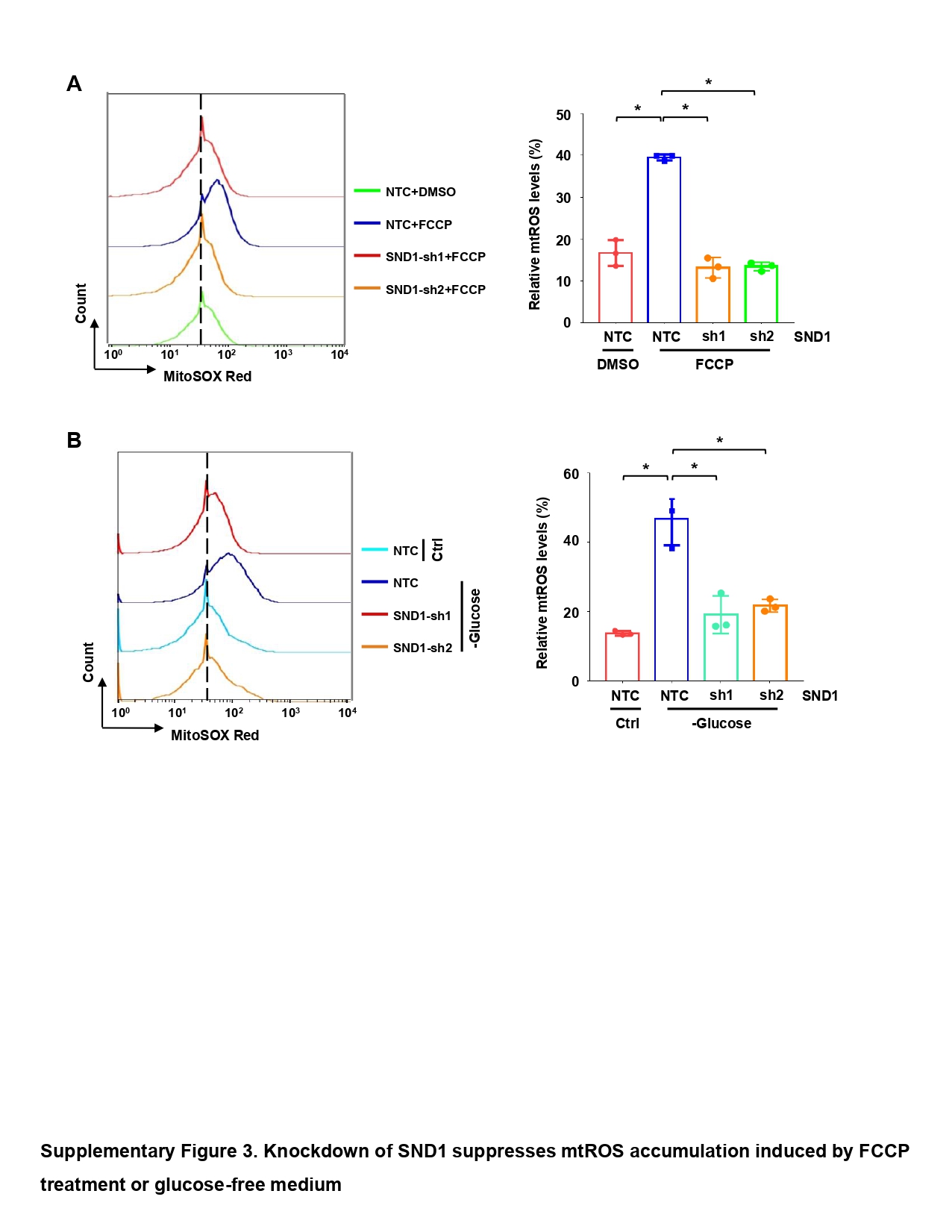

Supplement: Supplementary Figure 3 — Knockdown of SND1 suppresses mtROS accumulation induced by FCCP treatment or glucose-free medium. (A) Hep3B cells stably expressing shSND1 were treated with 10 μM FCCP for 6 h. Samples were collected and the mitochondrial ROS levels were analyzed by flow cytometry. *P < 0.05 comparing with the indicated groups. (B) Hep3B cells stably expressing shSND1 were treated with glucose-free medium for 24 h. Samples were collected and the mitochondrial ROS levels were analyzed by flow cytometry. * P < 0.05 comparing with the indicated groups. [file Image_3.jpg]
