# Supplementary material for: Mitochondrion-Localized SND1 Promotes Mitophagy and Liver Cancer Progression Through PGAM5
Source: Front Oncol. 2022 Mar 31;12:857968. doi: 10.3389/fonc.2022.857968 (PMC9008731; doi:10.3389/fonc.2022.857968)
Supplement: Supplementary file 4 [file Table_1.doc]

| **Supplementary Table 1: Construct primers** | |
| --- | --- |
| **Primer name** | **Primer Sequence** |
| pSin-3×Flag-hs-SND1-Fwd | AGGTGTCGTGAGGAATTCATGGCGTCCTCCGCGCA |
| pSin-3×Flag-hs-SND1-Rev | GTCATGGTCTTTGTAGTCACTAGTGCGGCTGTAGCCAAATTCGTC |
| pSin-3×Flag-hs-SND1^Δ1-63^-Fwd | AGGTGTCGTGAGGAATTCATGGCCACACAACCTGATGC |
| pSin-3×Flag-hs-SND1^Δ1-63^-Rev | TCATGGTCTTTGTAGTCACTAGTGCGGCTGTAGCCAAATTCGTC |
| pSin-3×Flag-hs-SND1-N-Fwd | AGGTGTCGTGAGGAATTCATGGCGTCCTCCGCGCA |
| pSin-3×Flag-hs-SND1-N-Rev | GTCATGGTCTTTGTAGTCACTAGTGTGGGCCCAGACCTTCTCTTTCT |
| pSin-3×Flag-hs-SND1-C-Fwd | AGGTGTCGTGAGGAATTCATGTATGAGGAGCAGCCCGTGGAG |
| pSin-3×Flag-hs-SND1-C-Rev | GTCATGGTCTTTGTAGTCACTAGTGCGGCTGTAGCCAAATTCGTC |
| pSin-HA-hs-SND1-Fwd | GCTAGCATCGATACGCGTATGGCGTCCTCCGCGCA |
| pSin-HA-hs-SND1-Rev | TGCGGATCCTTCGAACTAGTGCGGCTGTAGCCAAATTCGTC |
| pSin-3×Flag-hs-PGAM5-Fwd | AGGTGTCGTGAGGAATTCATGGCGTTCCGGCAGGCG |
| pSin-3×Flag-hs-PGAM5-Rev | GTCATGGTCTTTGTAGTCACTAGTGGATCGAGTGATCTTGTCGGGAG |
| pSin-3×Flag-hs-PGAM5-N-Fwd | AGGTGTCGTGAGGAATTCATGGCGTTCCGGCAGGCG |
| pSin-3×Flag-hs-PGAM5-N-Rev | GTCATGGTCTTTGTAGTCACTAGTCCGCGTGGCCTTGGCTTTGTA |
| pSin-3×Flag-hs-PGAM5-C-Fwd | AGGTGTCGTGAGGAATTCATGCACATCTTCCTCATCAGGCATTCCCA |
| pSin-3×Flag-hs-PGAM5-C-Rev | GTCATGGTCTTTGTAGTCACTAGTGGATCGAGTGATCTTGTCGGGAG |
| pSin-HA-hs-PGAM5-Fwd | GCTAGCATCGATACGCGTATGGCGTTCCGGCAGG |
| pSin-HA-hs-PGAM5-Rev | TGCGGATCCTTCGAACTAGT TCAGGATCGAGTGATCTTGTCGGGAG |
| pSin-3×Flag-hs-TOM20-Fwd | AGGTGTCGTGAGGAATTCATGGTGGGTCGGAACAGCG |
| pSin-3×Flag-hs-TOM20-Rev | GTCATGGTCTTTGTAGTCACTAGTTTCCACATCATCTTCAGCCAAGCT |
| pSin-3×Flag-hs-TOM70-Fwd | AGGTGTCGTGAGGAATTCATGGCCGCCTCTAAACCTGTGG |
| pSin-3×Flag-hs-TOM70-Rev | GTCATGGTCTTTGTAGTCACTAGTTAATGTTGGTGGTTTTAATCCGTATTTCTTTGC |
| pSin-HA-hs-DRP1-Fwd | GCTAGCATCGATACGCGTATGGAGGCGCTAATTCCTGTCATA |
| pSin-HA-hs-DRP1-Rev | TGCGGATCCTTCGAACTAGT CCAAAGATGAGTCTCCCGGATTTCAGC |
| pET-22(b)-His-PGAM5-Fwd | GATGATGATAAACATATGATGGCGTTCCGGCAGGCG |
| pET-22(b)-His-PGAM5-Rev | GTGGTGGTGCTCGAGGGATCGAGTGATCTTGTCGGGAG |
| pGEX-4T1-GST-SND1-Fwd | GTTCCGCGTGGATCCATGGCGTCCTCCGCGCA |
| pGEX-4T1-GST-SND1-Rev | GTCACGATGCGGCCGCTTAGCGGCTGTAGCCAAATTCGTC |
| MTS-Fwd | GAATTCATGTTGGCTACCAGGGTATTTAGCCTAGTTGGCAAGCGAGCAATTTCCAC  CTCTGTGTGTGTACGA |
| MTS-Rev | ACGCGTTCGTACACACACAGAGGTGGAAATTGCTCGCTTGCCAACTAGGCTAAATA  CCCTGGTAGCCAACAT |
