# Supplementary material for: Mitochondrion-Localized SND1 Promotes Mitophagy and Liver Cancer Progression Through PGAM5
Source: Front Oncol. 2022 Mar 31;12:857968. doi: 10.3389/fonc.2022.857968 (PMC9008731; doi:10.3389/fonc.2022.857968)
Supplement: Supplementary file 5 [file Table_2.doc]

| **Supplementary Table 2: Oligonucleotide sequences of shRNAs** | | |
| --- | --- | --- |
| **shRNAs** | **shRNA-ID** | **Sequence** |
| Non-Target Control | SHC002 | CCGGCAACAAGATGAAGAGCACCAACTCGAGTTGGTGCTCTTCATCTTGTTGTTTTT |
| Hs-SND1-sh1 | TRCN0000049656 | CCGGCGGGATCTCAAGTATACCATTCTCGAGAATGGTATACTTGAGATCCCGTTTTTG |
| Hs-SND1-sh2 | TRCN0000049657 | CCGGGCTGATGATGCAGACGAATTTCTCGAGAAATTCGTCTGCATCATCAGCTTTTTG |
| Hs-SND1-sh3'UTR | This study | CCGGTCTCTGTCCAACTGTTGATTA CTCGAG TAATCAACAGTTGGACAGAGATTTTTG |
| Hs-TOM70-sh1 | This study | CCGGGCATGCTGTTAGCCGATAAAGCTCGAGCTTTATCGGCTAACAGCATGCTTTTTG |
| Hs-PGAM5-sh1 | This study | CCGGGCCGGAAGCTGTGCAGTATTACTCGAGTAATACTGCACAGCTTCCGGCTTTTTG |
| Hs-PGAM5-sh2 | This study | CCGGGCCATAGAGACCACCGATATCCTCGAGGATATCGGTGGTCTCTATGGCTTTTTG |
